# Transvaginal rectocele repair with human dermal allograft interposition and bilateral sacrospinous fixation with a minimum eight-year follow-up

**DOI:** 10.1186/s12894-016-0135-9

**Published:** 2016-03-25

**Authors:** Serge P. Marinkovic, Scott Hughes, Donghua Xie, Lisa M. Gillen, Christina M. Marinkovic

**Affiliations:** Department of Urology, Detroit Medical Center, Harper/Hutzel Hospital, Detroit, MI 48202 USA

**Keywords:** Rectocele repair, Sacrospinous fixation, Human dermal allograft

## Abstract

**Background:**

Human dermal allografts have been used for over a decade for interpositional repair of rectoceles. How do dermal allografts perform with regards to success rate and complications with 8 years’ minimum follow-up?

**Methods:**

We retrospectively reviewed 41 consecutive patients undergoing dermal allograft interposition procedures between October 2001 and December 2005 (Repliform, Boston Scientific, Natick, MA, USA) for stage two, three, and four International Continence Society (ICS) symptomatic rectocele repairs with bilateral sacrospinous fixation. Failure was defined as recurrent stage two International Continence Society prolapse (Ap ≥ −1 and/or Bp ≥ −1). All questionnaires were completed 1 week before surgery and at follow-up (September 2014 through December 2014).

**Results:**

The mean preoperative and postoperative A(p) were 0.95 ± 0.70,−1.90 ± 0.52 and B(p) 1.30 ± 0.84,−2.13 ± 0.51 (*p* < 0.001). With a mean follow-up of 116.5 ± 18.9 months, a success rate of 73 % (30/41) was achieved, with anatomical reduction of prolapse. For splinting and digitations, an 82 % cure rate was realized. The Pelvic Floor Distress Inventory (PFDI) pre- and post-operative results showed significant improvement (*p* < 0.001). There were two incisional exposures (5 %). Seventy percent of patients were secondary repairs while 30 % were primary repairs (81 % success rate, *p* < 0.36). One patient experienced nerve entrapment and subsequent unilateral takedown. Patient satisfaction was 77 %.

**Conclusions:**

Our retrospective study approaching long-term results demonstrated that symptomatic rectocele procedures with human dermal allograft interposition provide an effective anatomical and functional repair with acceptable complication rates.

## Background

Pelvic organ prolapse is a common surgical women’s health care issue affecting over ten million American women, at a projected cost of two billion dollars (USD) each year [1]. Approximately 12 % of adult women will eventually require surgical therapy for their symptomatic prolapse [2]. Prolapse is the result of a multitude of molecular and physiological changes that cause weakening in one or more supportive structures in the pelvic compartment. Rectal protrusions are attributed to connective tissue defects in the rectovaginal fascia that are level two Delancey pelvic support mechanisms. The most commonly used surgical procedures for repair in this area involve suture ligation with native tissue plication [3]. Another method of rectocele repair is defect-specific re-approximation of the rectovaginal fascia without levator plication [4, 5]. This procedure results in less post-operative pain but not much improvement in reduced rectocele recurrence.

Practitioners have tended to agree that, in the instance of transvaginal organ prolapse repairs, patients’ innate connective tissue defects can be repaired with the interposition of biologic human allograft dermal material [6, 7] or synthetic materials such as polypropylene [8]. Today, these biologic materials [9] abound, yet long-term studies are lacking. Available studies have multitudes of clinical criteria for evaluation and measurements for failure, making comparisons inaccurate and difficult to assess. Clinical assessments are often retrospective and include a variety of surgical approaches, so clear-cut evaluations and critiques regarding usefulness remain unclear.

Synthetic materials categorized via the Amid [10] (1997) five-material classification system have been applied to what was originally used for general surgeons’ herniorrhaphy procedures and are now also applied to female pelvic prolapse surgeries (Table 1). Materials are separated into macroporous, microporous, or both, and include three well-studied materials: polypropylene, mersilene and polytetrafluoroethylene. In 2011, the Federal Drug Administration issued a stern warning against the transvaginal utilization of synthetic materials, prompting a voluntary removal of several pelvic floor reconstruction products from the market (i.e., Prolift, Gynecare, Somerset, New Jersey). As a result, surgeons were left with fewer options for transvaginal prolapse repairs. For more than 12 years, we have extensively used dermal allograft materials (Repliform, Boston Scientific, Natick, Massachusetts, USA) for symptomatic rectocele repairs with bilateral sacrospinous fixation [11–14] and now report our minimum eight-year follow-up experience with this human allograft product.

**Table 1:** Classification of Synthetic Meshes (Amid, 1997)[10]

Type 1: Totally Macroporous (pore size > 75 μ)
• Prolene • Gynemesh PS • Gynecare TVT • SPARC
Type 2: Totally Microporous (port size < 10 μ)
• Goretex
Type 3: Macroporous with Filaments or Microporous Components
• IVS • Uratape • Surgipro • Mersilene • Parietex
Type 4: Submicronic Pore Size (pore size < 1 μ)

## Methods

Between October 2001 and December 2005, we performed 41 consecutive symptomatic repairs with human dermal allograft interposition and bilateral sacrospinous fixation for stage two, three, or four International Continence Society rectoceles, with or without digitation and/or splinting. All rectoceles were performed with suture ligation of the new human dermal allograft fascia to the rectovaginal fascia with bilateral sacrospinous fixation. Surgeries were performed in their entirety by the first author. The Caprio device [11] (Boston Scientific, Natick, MA, USA) was used, with 0-polypropylene sutures (two sutures applied to the right sacrospinous ligament and another two to the left ligament). Failure was defined as recurrent International Continence Society stage two or more prolapses. All patients underwent a complete history and physical examination with International Continence Society prolapse (POP) scoring. Urodynamics were performed only if the patient was also undergoing a concomitant continence procedure performed with a tension-free vaginal tape (TVT, Gynecare, Somerset, New Jersey), tension-free vaginal tape obturator (TVTO, Gynecare, Somerset, New Jersey) or tension-free vaginal tape-Secur (Gynecare, Somerset, New Jersey, USA). Urodynamic results are not included in this study. All preoperative and postoperative data were collected between September 2014 and December 2014. Our research was performed in accordance with the Declaration of Helsinki and was approved by the Harper Hospital Institutional Review Board (Study #033512MP4E). The Pelvic Floor Distress Inventory-20 (PFDI) [15] and the 7 point Likert Visual Analogue Scale questionnaire were used to objectively determine patient satisfaction and improvement of symptoms following surgery. On the Likert Scale (Table 2), only levels of five, six and seven were recorded as satisfactory outcomes. Levels 1–4 were recorded as unsuccessful outcomes. All patients included their age, parity, body mass index, smoking status, menopausal, hormone therapy, and prior pelvic surgery. All exposures were treated the same, with two grams of topical estrogen thrice weekly for 6 weeks. Follow-up examinations were performed by an unaffiliated, board-certified gynecologist with 30 years of experience, who was well acquainted with POPQ staging and scoring. Statistical analyses were performed using the Statistical Package for Social Sciences, version 11.0 (SPSS, Chicago, Illinois). Analyses included simple means, medians, and chi square comparisons.

**Table 2:** Likert Global Response Visual Analogue Scale for Rectocele Repair

Since having your rectocele repair, please rate your overall rectocele symptoms:		
1.	Markedly Worse	1
2.	Moderately Worse	1
3.	Slightly worse	1
4.	Same	1
5.	Slightly improved	1
6.	Moderately improved	2
7.	Markedly improved	3

## Results and discussion

The mean preoperative and postoperative A(p) were 0.95 ± 0.70,−1.90 ± 0.52 and B(p) 1.30 ± 0.84,−2.13 ± 0.51 (*p* < 0.001) (Table 3). With a mean follow-up of 116.5 ± 18.9 months, a success rate of 73 % (30/41 patients) was achieved, with the anatomical reduction of prolapse to ICS Stage 2 or less (Ap ≥ −1 and/or Bp ≥ −1). For splinting and digitation elimination, an 82 % cure rate was realized (15/18). In the follow-up period, 13 % (3/23) of patients complained of de novo splinting. First-time repairs demonstrated an 81 % anatomical success rate while secondary repairs detailed a 70 % anatomical repair. Seventy percent were secondary repair, with a 70 % success rate while 30 % of the patients were primary repairs (81 % success rate) (*p* < 0.36). The Pelvic Floor Distress Inventory (PFDI) pre- and postoperative results showed significant improvement (*p* < 0.001). There were three incisional exposures (7 %) of the human dermal allograft, which responded to estrogen replacement therapy for 6 to 8 weeks. One patient experienced rectal outflow obstruction and another pelvic nerve entrapment, which caused left gluteal pain. Both required re-operative take down at two and twenty weeks after rectocele repair. Patient satisfaction was 77 % (Table 3). De novo dyspareunia was 17 %.

**Table 3:** Patient Demographics (*n* = 41)

Mean Age (yrs)	60.6 ± 16.3
Mean Follow-up (months)	116.5 ± 18.9
Mean Parity	2.6 ± 2.37
Mean Body Mass Index	34.4 ± 6.1
Smoker (percent)	34.1
Menopausal (percent)	65.3
Post-Hysterectomy (percent)	41.4
Hormone Replacement (percent)	40.9
Sexually active (percent)	24.1
Previous Pelvic Surgery (percent)	70.0
Mean Preoperative A (p)(centimeters)^a^	0.95 ± 0.70
Mean Postoperative A (p) (centimeters)^a^	−1.90 ± 0.52
Mean Preoperative B (p)(centimeters)^a^	1.30 ± 0.84
Mean Postoperative B (p)(centimeters)^a^	−2.13 ± 0.51
Mean PreOp PFDI-20 (/300)^a^	129.6 ± 26.7 (78–223)
Mean PostOp PFDI-20 (/300)^a^	60.9 ± 18.4 (32–108)
Biological erosion (percent)	7.3
De novo dyspareunia (percent)	17.0
Complication rate (percent)	12.1
Patient satisfaction (percent)^b^	77.0
Prolapse Failure Rate (percent)^c^	27.0
Time to Prolapse Failure (months)	24.7 ± 15.3

^a^Changes between pre- and postoperative Pelvic Floor Distress Inventory (PFDI-20, *n* = 41) and A(p) and B(p) were statistically significant at *p* = <0.001 at the time of patients’ last follow-up

^b^A 7 point Likert Global Response Scale was used, whereby responses five, six and seven were deemed successful. Numbers 1–4 were judged as failures. The 77 % satisfaction rate includes only the former and not the latter

^c^Eleven prolapse failures: 7 Stage 2 (ICS), 3 Stage 3, and 1 Stage 4

A review of rectocele repairs shows that, for a host of reasons, procedures without augmentation have not been successful. Pelvic floor remodeling of the supportive connective tissue continues to occur, with increased activity of collagenase and elastase enzymes [16, 17]. Elastase degradation leads to decreased connective tissue flexibility and expansion [18]. Together, these biologically enzyme processes lead to a potentially weaker pelvic floor infrastructure [18].

Native tissue amended with suture ligation and/or fixation to autologous ligaments or bone may lead to compromised clinical results because of the continual exposure to connective tissue remodeling [19–22]. Surgical treatment has seen the development of many transvaginal synthetic pelvic floor prolapse repair kits [23]. These kits have not fared as well as transvaginal stress incontinence instruments and materials, which are made of similar synthetic materials but with a more limited exposure area. [24] While stress incontinence kits are not exempt from failure or complications including exposure or erosion into the vagina or pelvic organ, in the past 5 years, prolapse repair kits have found themselves at the forefront of malpractice litigation. In the early 2000’s several studies with the biological xenograft (porcine) product Pelvichol [25] (C.R. Bard, Inc. Murray Hill, New Jersey, USA) demonstrated hastened reabsorption by the body and left pelvic floor surgeons much less enthusiastic towards its use. However, experience with human dermal allograft in 2000 gave us adequate two-year and four-year data for rectocele repairs [5, 6] while we still used abdominal sacrocolpopexy for all multi-compartment prolapse repairs. Approaching 10 years’ follow-up (116.5 ± 18.9 months), a good success rate can be attributed to a lasting biological material and augmentation of the posterior pelvic floor compartment with a concomitant bilateral sacrospinous fixation with permanent suture. Limitations of our study included two major complications: the unilateral takedown of the rectocele repair/augmentation because of rectal outflow obstruction, and pelvic nerve entrapment, which required a unilateral takedown. These complications combined with three vaginal exposures gave us a complication rate of 12 %. Both patients were morbidly obese, less than five feet tall, with body mass index scores of 40 and 41.

The 11 patients who failed their surgery had a mean time to failure of 24.7 ± 15.3 months. At the time of their sacrocolpopexy, we carefully examined the pelvic compartment for the human dermal allograft. Quantities of tissue were still found circumscribing the periphery of the rectovaginal fascia, but the attachment to the polypropylene suture had, in most cases, pulled through from the sacrospinous ligament and/or the tissue was tattered in appearance. Of these 11, eight elected to have surgery to repair their rectoceles, but because the prolapse now involved more than one compartment, six of the eight elected to undergo sacrocolpopexy with anterior and posterior polypropylene mesh interposition.

Encouraging factors for the utilization of human dermal allograft include its good anatomical reduction of the rectocele, as well as symptom improvement with a follow-up in many cases exceeding 10 years. This efficacy can be considered a driving force for its continued utilization in the posterior compartment symptomatic rectocele repair; however, Level 1 and/or Level 2–1 evidence studies should be conducted to better compare the efficacy and safety of human dermal allograft to other pelvic floor materials.

## Conclusions

Our retrospective study demonstrated that rectocele repairs with biological augmentation and bilateral sacrospinous fixation with a minimum 8 years’ follow-up provide a good anatomical and functional repair with an acceptable complication rate.

### Brief summary

Human dermal allograft interposition repair of rectoceles can be used safely and successfully, with good patient satisfaction in follow-up periods approaching 10 years.
